# Microstructure Characterization of Reversed Transformation in Cryogenically Rolled 22MnB5

**DOI:** 10.3390/ma13071741

**Published:** 2020-04-08

**Authors:** Shengjie Yao, Long Chen, Guannan Chu, Hongyun Zhao, Lei Feng, Guodong Wang

**Affiliations:** 1 School of Materials Science and Engineering, Harbin Institute of Technology at Weihai, Weihai 264-209, China; 19955369069@163.com (L.C.); chuguannan@163.com (G.C.); hy_zhao66@163.com (H.Z.); wanggd@ral.neu.edu.cn (G.W.); 2 Shandong Provincial Laboratory of Special Welding Technology, Harbin Institute of Technology at Weihai, Weihai 264-209, China; 3 Qingdao Automotive Research Institute of Jilin University, Qingdao 266-108, China; 15689102728@163.com; 4 State Key laboratory of Rolling Technology and Automation, Northeastern University, Shenyang 110-009, China

**Keywords:** asymmetric rolling, phase transformation, recrystallization, quenchable steel

## Abstract

Hot stamping is a well-known process to produce structural automotive parts with an excellent strength-to-weight ratio. However, this process is more expensive due to the lower energy efficiency and operating cost of the traditional roller-hearth furnace. Additionally, lower ductility and toughness are commonly recognized as the main disadvantages of the current hot stamped ultra-high-strength parts. Refinement of austenite grains could be a profitable way to improve the strength of hot stamped parts. In this work, the evolution of reversed transformation in asymmetrically cryogenically rolled samples was studied in order to control the austenite. Thermomechanical simulation and heat treatment in the salt bath were used to investigate the reversed transformation process, and the typical microstructures were characterized by transmission electron microscopy (TEM) and scanning electron microscopy (SEM). Compared with symmetric prerolling, ferrite recrystallization could be remarkably inhibited by asymmetric rolling at the liquid nitrogen temperature (LNT) during the reheating process. Additionally, the nucleation of the austenite inner grains can also be promoted and the dynamics of the reversed transformation accelerated by asymmetric prerolling. Such phenomena might be very useful to refine the parent austenite grains before press hardening and enhance the new hot stamping strategy by partial fast reheating.

## 1. Introduction

The use of high-strength steel sheets for automobile parts has remarkably increased as a result of weight reduction and safety demand. In particular, quenchable steels (typically 22MnB5), one of the most popular representatives, have been increasingly used in the hot stamping of ultra-high-strength steel parts for automobile manufacturing since it was first used as side impact door beams in the SAAB 9000, which went into production in 1984 [1]. As of 2016, the use of hot formed steel rose to a higher level in the Volvo XC90, accounting for 38% of the body mass [2]. Similarly, the strength of hot stamped parts has gradually increased due to the strong demand for cars with higher intrusion resistance and lighter body frame. For example, the quenched tensile strength of 27MnCrB5, 28MnB5, 34MnB5 and 37MnB4 has reached to 1611, 1740, 1919 and 2040 MPa, respectively [3,4].

The reason for the popularity of the above-mentioned quenchable steels is mainly attributed to its transformation from austenite into its fully martensitic crystal structure during press hardening. Therefore, the characteristic of reverted austenite is of critical importance to the final microstructure and property of hot stamped parts. It is also well established that the reverse transformation behavior is sensitive to the initial microstructure [5], which for the Mn-B alloyed steels is currently delivered in mostly soft ferritic-pearlitic condition.

There are numerous studies on the reverse transformation from a pearlitic structure or ferrite/cementite structure [6,7,8]. Theoretically, it is accepted that austenite preferentially nucleates at the pearlite colony boundary and at the ferrite/cementite interface inside a pearlite colony [9], especially at the high angle boundary (HAB) of the ferrite in pearlite [8]. However, the reverse transformation is complicated due to the inhomogeneously distributed carbon and alloying elements, which can be equilibrated at the interface for interstitial alloying elements [10], or negligible-partition local equilibrium [11]. In particular, more and more alternative heating technologies are being used in the hot stamping process, such as induction heating [12], conduction heating [13] and direct contact heating [14], in order to improve production efficiency by shortening the process cycle time. Li et al. studied the effects of thermal conditions on austenite formation in a 22MnB5 manganese-boron steel with an initial microstructure composed of a mixture of approximately 78% proeutectoid ferrite and 22% pearlite [15]. They found that for a larger amount of austenite formation the heating rate effects were greater on the temperature than on time, and such effects could be helpful to enhance productivity and reduce energy consumption in the hot stamping process.

Additionally, Bettanini et al. suggested that Cr-rich carbides control the austenite transformation kinetics due to Cr partitioning and stabilization of ferrite, and coarse carbides and fast heating rate lead to a soft-impingement of the Cr diffusion fields ahead of austenite interface [16]. Lolla et al. actually found a high-Cr interface concentration at the cementite/austenite interface during the continuous heating process, and the interface velocity was controlled by Cr-diffusion [17]. Based on such findings, they took advantage of the rapid heating and short hold-time, together with undissolved carbides, in order to prepare ultrafine grained austenite with carbon inhomogeneity, and subsequently a mixed microstructure during rapid cooling.

In addition, Takafumi et al. systematically investigated the effects of the heating rate, heating temperature, material conditions and cold rolling reduction on austenite nucleation and grain refinement in boron steels [18]. They found that the initial microstructure with bainite was more helpful to increase the effective grain boundary length for austenite nucleation per unit area than was with ferrite/pearlite. They also observed that the austenite distribution becomes more uniform, indicating that more potentially reverted nuclei could be activated by predeformation. A study by Yan et al. revealed that the reversed austenite tended to be completely granular when transformed from a cold-rolled martensite in a medium Mn steel, whereas acicular and granular austenite were reversely transformed from as-quenched martensite [19]. Han et al. [20] and Yang et al. [21] additionally reported that the reverse transformation mechanism changed from diffusive to diffusive massive transformation when the heating rate was above 15 °C/s.

Tokizane et al. [22] and Yang et al. [23] studied the formation of austenite in deformed lath martensitic and tempered the martensitic structure of low carbon steels. The competition between the recrystallization of the matrix and the formation of austenite occurred during the reheating process in samples with different cold rolling reductions.

However, it remains unknown, what happens when the predeformation is implemented by asymmetric rolling after the sample is cooled in liquid nitrogen, which can change both dislocation density and grain orientation in the initial microstructures. Usually, many medium and low stacking fault energy (SFE) metals are mainly considered in the cryo-rolling process, such as copper alloys, aluminum alloys and some fcc-structured steels, as the dynamic recovery can be easily suppressed and even the deformation twinning can be stimulated, which always promotes further grain refinement and strengthens the materials [24,25,26]. For bcc-structured ferrite, even though the fast recovery will greatly change the dislocation density during cryo-rolling, the effect on the ferrite-to-austenite transformation behavior is still unclear as an initial microstructure before the reheating process. Bellavoine et al. [27] noted that incomplete recrystallization led to the acceleration of austenite formation, and the overlap of ferrite recrystallization and austenite formation in the case of high-heating rate resulted in a higher density of austenite nuclei in cold-rolled advanced high-strength steels.

Therefore, the objective of this study was to investigate the reverse transformation behavior as a function of cryo-rolling reduction and linear velocity rate, using commercially available 22MnB5 steel. With that clarification, ultrafine grained austenite could be more industrially practicable, and then, it could be helpful to propose a new promising hot stamping process for manufacturing higher strength parts with much lower cost, since work-hardened 22MnB5 will be used without any addition of chemical compositions, such as niobium, titanium and/or molybdenum.

## 2. Materials and Methods

**Figure 1:**
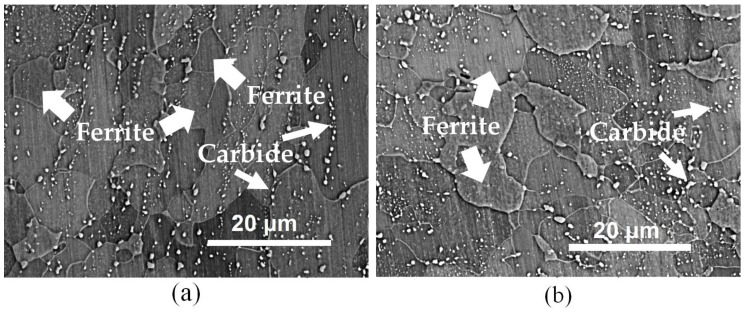
Scanning electron microscopy (SEM) microstructure observed from different locations of the as-received 22MnB5 steel (**a**) cross section and (**b**) rolling surface. Ferrite and dispersed carbide are mainly discovered in the as-received steel.

The chemical composition of commercially available 22MnB5 steel sheets is listed in Table 1. The sheets with initial thickness of 1.8 mm had an initial microstructure of ferrite and carbide, as shown in Figure 1. The critical transformation temperature of the 22MnB5 steel was *A*_3_ = 811 °C and *A*_1_ = 736 °C [28].

**Figure 2:**
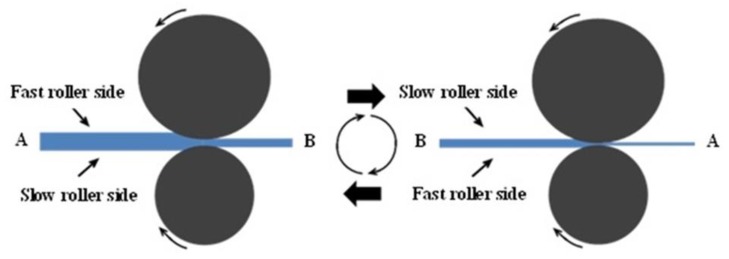
Schematic diagram of the asymmetric rolling process. Two passes, for example: the first pass is implemented as the left picture, and the second one proceeds after the sample flipped front and rear, simultaneously up and down as shown in the right picture.

Before the reverse transformation experiments, the sheets were first asymmetrically cryo-rolled in a non-reversing mill with a pair of ladder rollers at ambient temperature. In particular, the asymmetric cryo-rolling method performed an even number of passes, in which the rolling material was simultaneously rolled by turning the rolling material upside down and by changing directions of the rolling material. Before each rolling pass, the specimen was cooled in liquid nitrogen for 5 min. The asymmetric rolling process is illustrated in detail in Figure 2. The selected rolling reductions of 30% and 50% corresponded to sheets of final thickness of 1.26 and 0.90 mm, respectively. The velocity ratio, referred to here as asymmetry ratio (AR), of the top and bottom roll speeds was changed by alternating the diameter difference between the two work rolls. In our experiment, the AR was chosen as 1.0, 1.2 and 1.5.

**Figure 3:**
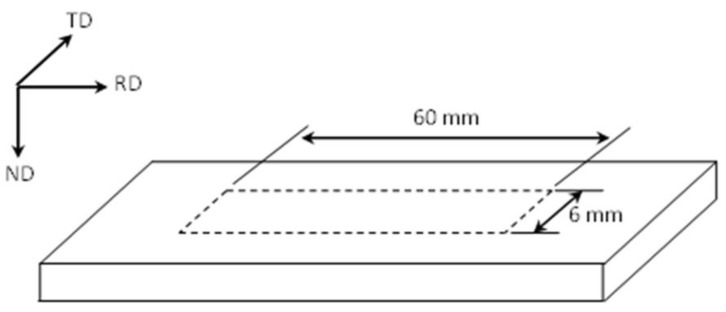
Sampling position and size in the cryogenically rolled sheet.

Thin specimens of 60 × 6 × *h* mm^3^ in size were cut by numerically controlled wire cutting machine from the cold rolled specimens along rolling direction for the reverse transformation (Figure 3). The phase transformation temperatures and dilation strains during continuous heating were measured using a dilatometer in an MMS-200^®^ Thermal Simulation Testing System (The State Key Laboratory of Rolling and Automation, Northeastern University, Shenyang, China). Dilatometry specimens were heated to their annealing temperature at various heating rates between 5.0 and 60 K•s^−1^ and then cooled to room temperature (RT) at a cooling rate of 30 K•s^−1^. 

In order to understand the intermediate microstructures before the austenite was fully transformed, some specimens were also heated in a salt bath at temperatures of 750 and 850 °C with holding times of 20 and 30 s, respectively, followed by water quenching.

The microstructures of the specimens were examined by field emission scanning electron microscopy (FE-SEM) using a JSM-6390 (JEOL Ltd., Tokyo, Japan) operated at 20 kV, equipped with an electron back-scattered diffraction (EBSD) and by field-emission transmission electron microscopy (FE-TEM) using a JEM-2100 (JEOL Ltd.) operated at 200 kV. Specimens of 15 × 10 × *h* mm^3^ for SEM were mechanically polished and etched in a 4% nital solution. The EBSD measurement was carried out with a TSL orientation imaging microscopy (OIM) system (TexSEM Laboratories, Inc., Provo, UT, USA). The step size chosen was 0.25 μm and the accelerating voltage was 25 kV. Thin foil specimens for TEM observations were mechanically ground to 50 μm in thickness and then pressed to prepare round disks of 3 mm in diameter. The disks were electropolished in an 8% perchlorate alcohol solution at −29 °C using a twin-jet polisher.

## 3. Results and Discussion

### 3.1. Characterization of Cryogenically Rolled Microstructure

**Figure 4:**
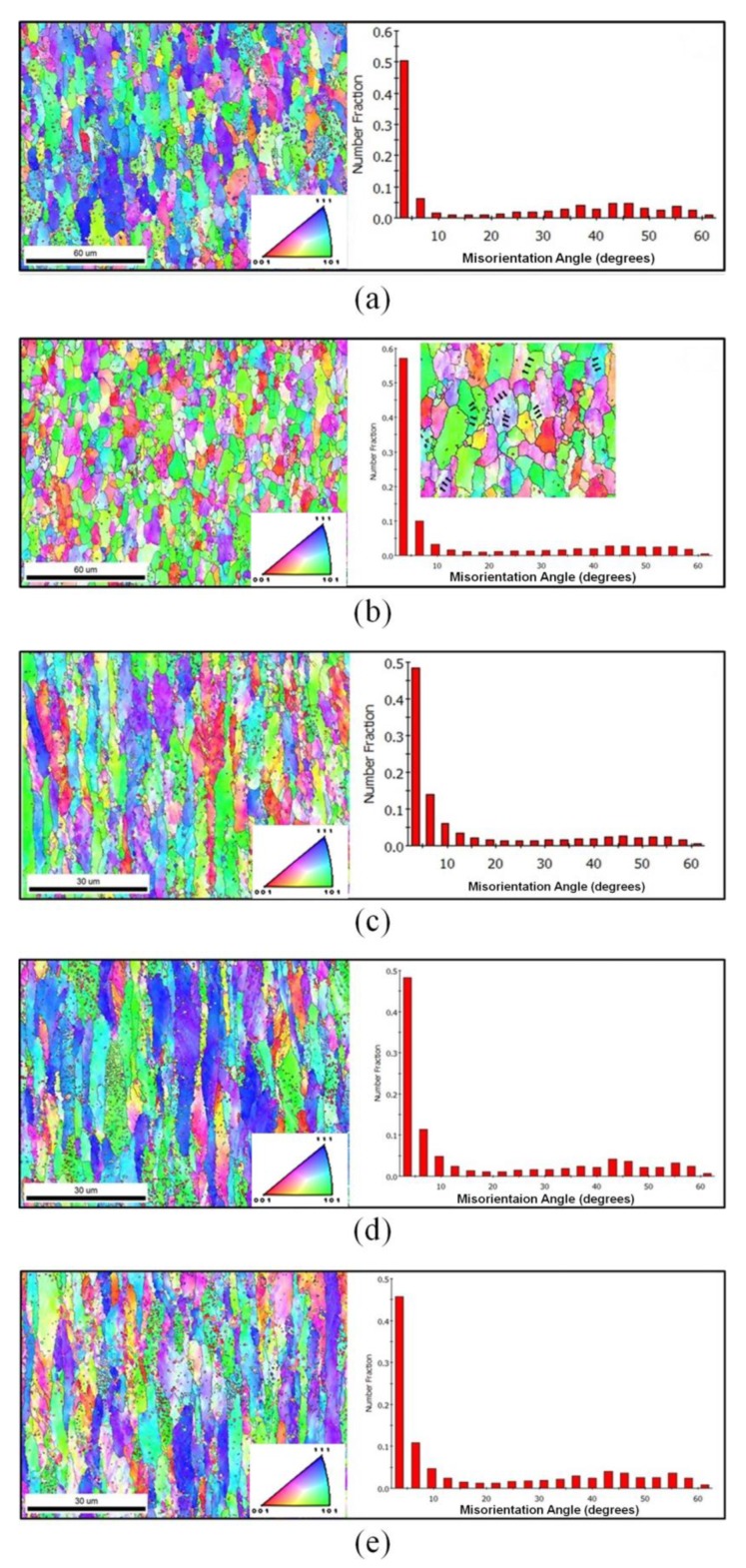
The electron backscatter diffraction (EBSD) map and misorientation angle distribution in samples with a total reduction of 30% (**a**, **b**) and 50% (**c**–**e**) rolled with an asymmetry ratio (AR) = 1.0 (**a**, **c**), AR = 1.2 (**b**, **d**) and AR = 1.5 (**e**).

The EBSD map and distribution of misorientation angles presented in Figure 4 show that the low angle grain boundaries (LAGBs) significantly increased with the increase of the AR from 1.0 to 1.2 for the samples with rolling reduction of 30%. In general, this will prevent grain growth and, by hindering the redistribution of dislocations at very low temperature, will contribute to an increase in their density and the development of the corresponding internal stresses [29]. Furthermore, asymmetric rolling can always greatly enhance the total strain applied to the materials. For the first pass, the applied strain can be larger due to the free-dislocation in the initial microstructure, and it can easily permeate across the thickness.

**Figure 5:**
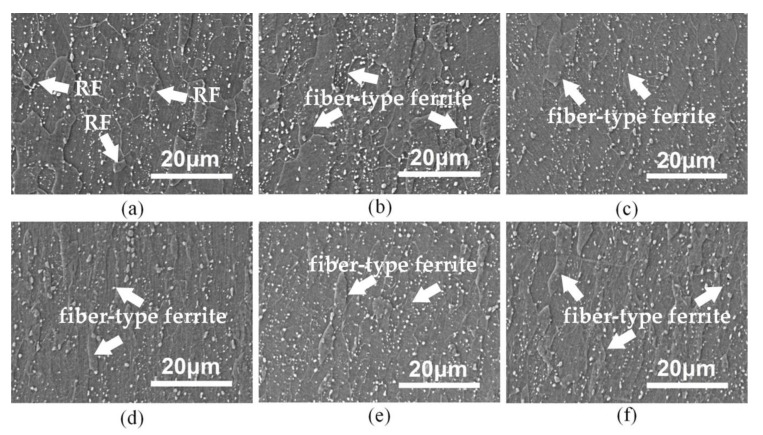
Microstructures of cryogenically prerolled samples with reduction of 30% (**a**–**c**) and 50% (**d**–**f**). Meanwhile, the AR is different as (**a**, **d**) AR = 1.0, (**b**, **e**) AR = 1.2 and (**c**, **f**) AR = 1.5.

As was well established, there was always a cross shear zone in the deformation zone when the elongation coefficient was larger than the AR, which was very helpful to increase the dislocation density and internal stresses. As the inset in Figure 4b shows, many low-angle misorientations could be clearly distinguished in some deformed ferrite grains with similar crystal orientation. However, it should be noticed that when the samples were rolled by 50%, the increase of the AR led to a significant decrease of the LAGB (<15°), which was indicated with the misorientation angle distribution shown in Figure 4c,d. Moreover, shear strains were clearly imposed with increasing the AR, and some intersecting shear deforming bands can be identified with the intragranular misorientation distribution images. This phenomenon could be greatly attributed to the asymmetric rolling method by rotating the sheet between passes. Meanwhile, two specific fiber textures <110>//RD and/or <111>//ND were developed when imposing shear strains by asymmetric rolling with higher reduction. The comparison of the microstructures of cryogenic prerolled samples shown in Figure 5a–c reveals that there were many nearly equiaxial recrystallized ferrite (RF) grains in the samples that were symmetrically rolled with a reduction of 30%. However, when the predeformation was performed by asymmetric rolling the deformed fiber-type ferrite remained in the samples. As mentioned above, the follow-up reduction in the pass became smaller and smaller, as a result of more and more strain hardening. When the elongation coefficient was smaller than the AR, the cross-shear effect could only be activated nearby the rolling surface. Although the SFE could be lowered by cooling at the liquid nitrogen temperature (LNT), according to a recent report by Mallick et al. [30], the severe shear-strain-induced temperature rise near the rolling surface could hardly delay the recovery process.

As shown in Figure 5d–f, all the ferrite remained in a non-recrystallized type with a reduction of 50%. Instead, the volume fraction of carbides changed with the AR. When the AR = 1.2, the volume fraction of carbides clearly increased and most of the carbides precipitated on the ferrite grain and subgrain boundaries, which could consume the LAGBs that arose from the rapid recovery of dislocations in ferrite grains. With the increase of the AR to 1.5, the volume fraction of carbides partly decreased and their size clearly increased, which indicates that further consumption of LAGBs could be partly achieved through the merging and growth of carbides. The precipitation of carbides inside the grains always requires proper nucleation sites, such as a subgrain boundary and other semicoherent interfaces.

**Figure 6:**
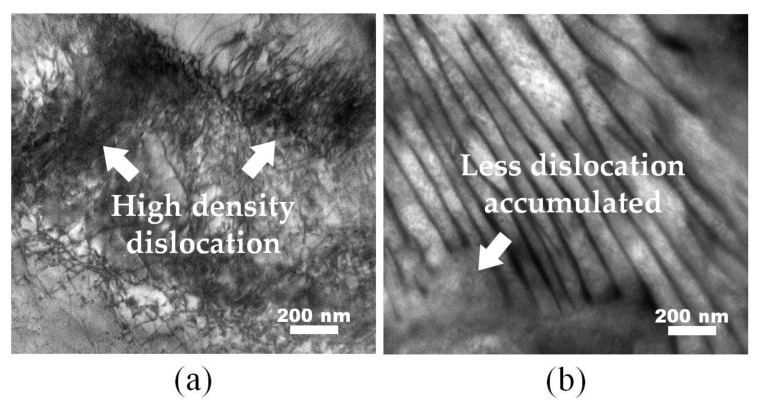
Transmission electron microscopy (TEM) morphology analysis of samples in reduction of 50% with AR = 1.5 at ambient temperature (**a**) and liquid nitrogen temperature (LNT; **b**).

However, the TEM images of the samples asymmetrically prerolled at different temperatures, shown in Figure 6, revealed that predeforming at ambient temperature produced more density of dislocations than that at the LNT. By measuring the yield strength of those samples, the dislocation density can be probably obtained with the empirical formula as follows:

(1) $$\Delta \sigma_{v}=\alpha Gb\xi^{1/2}$$

where Δσ_v_ is the yield strength difference, *α* is strength coefficient, *G* is the shear modulus, *b* is the Burgers vector and *ξ* is dislocation density. The result shows that the dislocation density of cryogenically rolled sample should be less than the cold-rolled one by 1.78 × 10^2^ times. Possible reasons for this include the high stacking fault energy of ferrite, and that the recovery can be more easily started after cryogenic rolling and fewer dislocations remain when the temperature of the sample returns to RT during the pass intervals. It is also inferred that some other kinds of defects (like vacancies) could be generated by cryogenic rolling. Thus, further investigation should be undertaken in order to clarify the micromechanism.

### 3.2. Thermal Dilation Curves of Reversed Transformation

**Figure 7:**
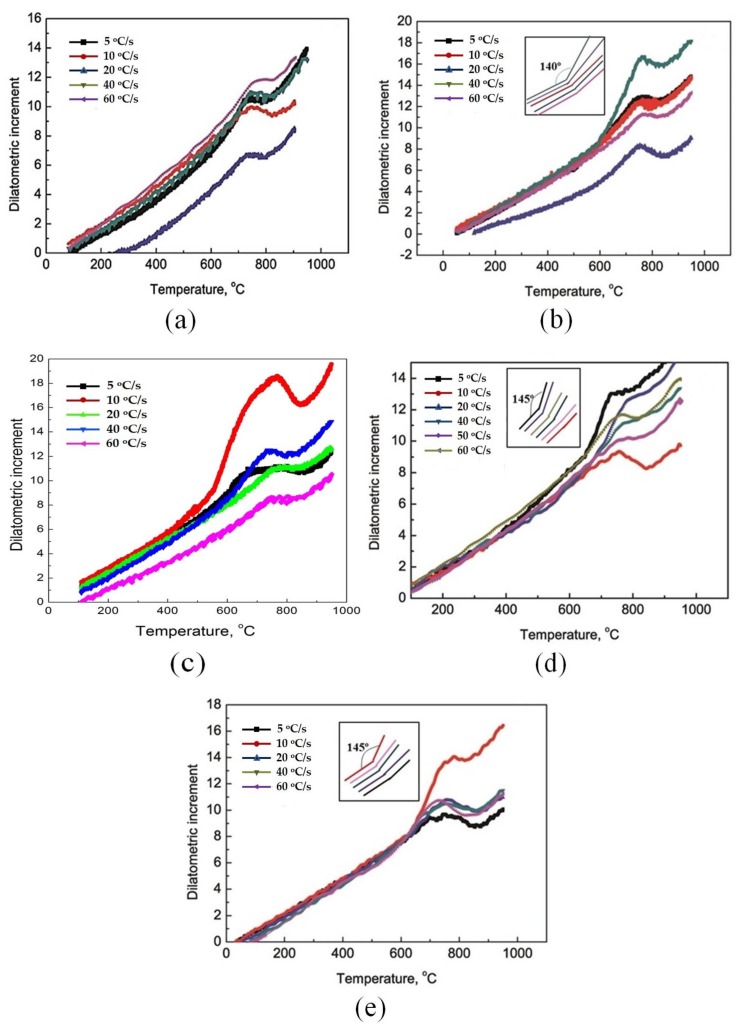
Dilation curves of the reversed transformation for different samples, wherein (**a**–**c**) show curves for samples prerolled with a reduction of 30%, 50% and 70% (AR = 1.2), respectively, (**d**, **e**) show curves for samples with reduction of 50% and with AR of 1.0 and 1.5, respectively.

For specimens with less prerolled deformation (30%), the changing gradients of all the dilation curves shown in Figure 7a were similar and consecutive before austenite transformation. This means that various kinds of microstructural evolution occurred during the reheating process, such as ferrite recovery, recrystallization, dissolution and/or precipitation of carbides. In Figure 7b, the slope of the dilation curve shows a sudden increase at the heating rate of 40 °C/s, while the other curves for the 50% predeformed samples had a very similar evolutionary trend, which was clearly shown in the inset. Remarkably, as the asymmetric prerolling deformation increased further, the sharp change of the slope tended to appear in the curve at the slower heating rate. As shown in Figure 7c, when the prerolling reduction increased to 70% with the same AR of 1.2, the slope was significantly increased in the curve at the heating rate of 10 °C/s. Additionally, all the sudden turning points were located at temperatures between 550 and 600 °C. It was also inferred that all these sudden slope changes were mainly introduced by ferrite recrystallization. After that, the process was dominated by dissolution of carbides, which can be clearly observed in Figure 7c. Moreover, the duration time of the carbide dissolution gradually decreased with increasing heating rate.

Considering the effect of the AR on the dilation curves, some regular results could be obtained by comparing Figure 7b,d,e. First, an abrupt increase in the slope of the dilation curves could be observed in all the three figures, even when the AR was set as 1.0. Second, the corresponding heating rate of those curves with a sudden slope change increased with the increase of the AR. Additionally, the slopes after the change remained nearly the same in the curves for all the samples processed by asymmetric prerolling, and they were a little smaller than that for samples processed by symmetric prerolling. Moreover, the dilation increase and temperature span after the sudden slope change before austenite transformation of asymmetrically prerolled samples were larger than those for symmetrically prerolled samples. Those inset graphs in Figure 7b–e corresponding to the transitional segments in different dilation curves show direct evidence for the above-mentioned viewpoint.

It was well established that the main driving force of microstructure transformation for deformed metals was the strain energy, where the stored energy from lattice distortion accounted for about 80%–90%. When the final deformation was in the ferrite region, numerous crystal defects would be introduced. Then, the nucleation rate of the second phase would be improved, thereby promoting the precipitation of microalloying elements. Meanwhile, for microstructure of ferrite and cementite prerolled at the LNT, the recovery proceeded more easily and was accompanied by precipitation of microalloying elements at the preliminary reheating stage, which is also shown in the inset of Figure 4b. There were many LAGBs (indicated by black arrows) distributed in larger deformed ferrite grains with similar crystal orientation. With increasing cryogenic rolling reduction, the amount of precipitation and the density of recovery boundaries were greatly improved, and that might delay the process of ferrite recrystallization, as illustrated in Figure 7.

**Figure 8:**
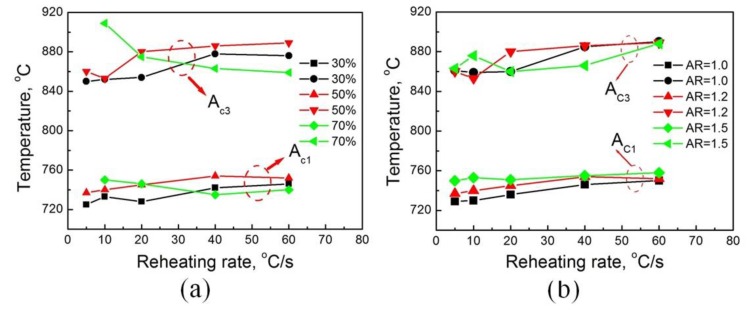
Dependence of *A*_c1_ and *A*_c3_ at different reheating rates on the rolling reduction (**a**) and AR (**b**), wherein AR equaled to 1.2 for (**a**) and the rolling reduction was chosen as 50% for (**b**).

In addition, prerolled deformation and the AR also have effects on the thermodynamics and kinetics of austenite transformation according to the results presented in Figure 8. As the reduction increases to 50%, the starting of the reversed transformation (A_c1_) was delayed, particularly when the reheating rate was lower than 20 °C/s, and this phenomenon agreed well with the results reported by Takafumi et al. [18].

However, the nucleation of austenite grains was more easily activated in the samples with a reduction of 70%. Meanwhile, both prerolling deformation and the AR in asymmetric rolling were beneficial to accelerate the kinetics of austenite transformation. Since little dislocation was found in the cryogenically rolled samples in Figure 6b, it can be inferred that many vacancies were generated during the deformation. Su et al. [31] reported that the dominant defects in cryogenically rolled aluminum were bulk monovacancies instead of vacancies associated with dislocations. Additionally, the higher concentration of bulk monovacancies inside the grains of the cryogenically rolled sample creates a larger driving force for vacancy annihilation to grain boundaries, which can also be a great driving force for the diffusion of carbon atoms during the reversed transformation, and contributes to the thermodynamics and kinetics. Moreover, the large amount of precipitation plays an important role in promoting the nucleation of reversed austenite grains.

### 3.3. Characterization of Reversed Microstructure Evolution

**Figure 9:**
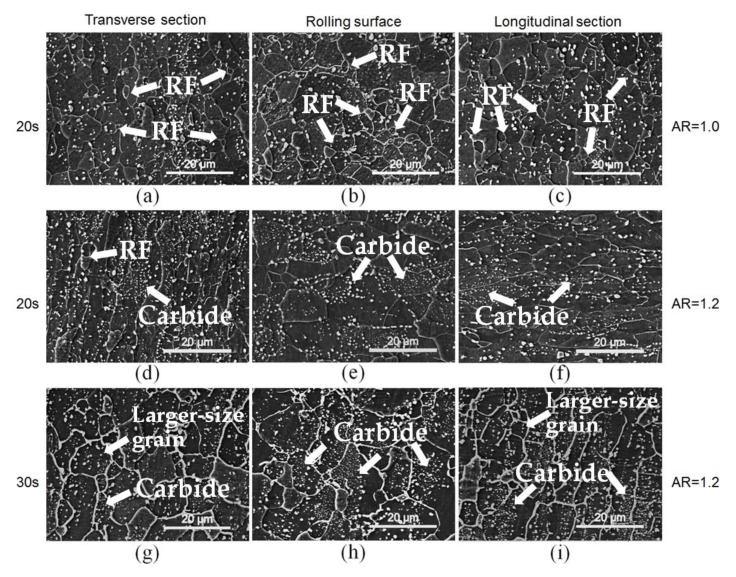
Scanning electron microscopy (SEM) images of the microstructures of sample prerolled with reduction of 30% and heat treated at 750 °C for 20 s (**a**–**f**) and 30 s (**g**–**i**), wherein (**a**–**c**) AR = 1.0 and (**d**–**i**) AR = 1.2.

The samples shown in Figure 9 were symmetrically and asymmetrically prerolled with reduction of 30% and AR of 1.2 and reheated in a salt bath at a temperature of 750 °C for 20 and 30 s. For symmetrically prerolled samples, shown in Figure 9a–c, the recrystallization of ferrite grains can be easily observed in all the sections with a holding time of 20 s. In comparison, few RF grains were observed in the asymmetrically prerolled samples (AR = 1.2) subjected to the same reheating process in Figure 9d–f. However, the volume fraction of carbides was significantly increased from 3.15% to 5.60%, and the size of the carbides is much smaller (105 nm) than that in symmetrically prerolled samples (260 nm). When the holding time was extended to 30 s, the microstructure in asymmetrically predeformed samples was typically RF and most of them were found in larger-size grains formed via grain growth. Instead, most of the remaining carbides were located in ferrite grains as shown in Figure 9g–i.

**Figure 10:**
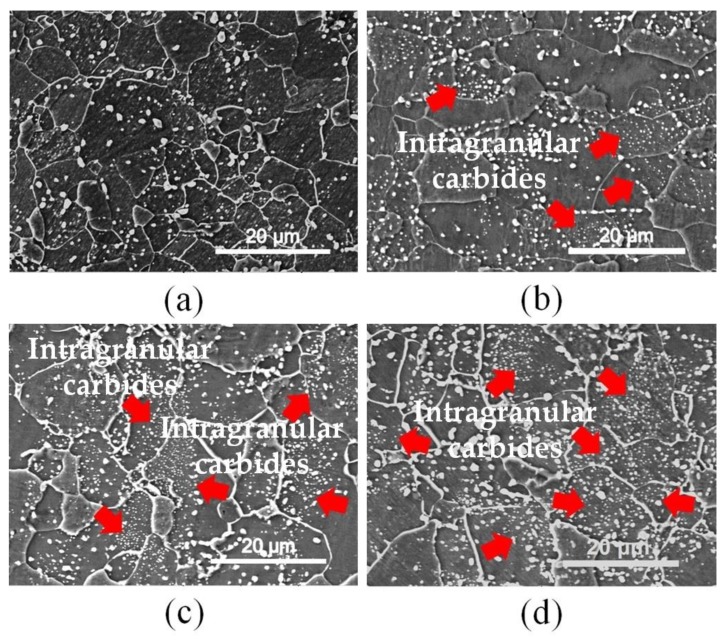
Morphology of carbides in the rolling surface of samples prerolled with reduction of 30% after being reheated at 750 °C for 20 s (**a**, **b**) and 30 s (**c**, **d**), wherein (**a**) AR = 1.0, (**b**, **c**) AR = 1.2 and (**d**) AR = 1.5.

When the investigation focused on the rolling surface where the asymmetric rolling features could be easily inherited in the microstructures, the effect of prerolling and holding time at certain reheating temperature on carbide morphology could be clearly observed, as shown in Figure 10. Notably, the amount of intragranular carbides in the rolling surface increased considerably with the increase of the AR and holding time at 750 °C, and most of the precipitated carbides were greatly refined to a nanoscale, as indicated by the red arrows in Figure 10b–d. Additionally, the reversed transformation was also affected by the evolving behavior of this microstructure since some thermodynamics features had changed before austenite transformation.

In Figure 10b,c, nanosized cementite abundantly precipitated in the interior of the grains, and the precipitation-free zones were clearly distinguished by transgranular boundaries in some larger ferrite grains. As Tamimi et al. concluded [32], the induced shear deformation in asymmetric rolling will result in texture evolution and may also affect microstructural features. Regarding the typical texture in a bcc-structure metal, it is always dominated by the rotated cube textures, such as {111} <110> and {001} <110>. Compared with the enlarged misorientation angle distribution shown in the image in Figure 4b, it can be inferred that certain crystal orientation is induced through cryogenic asymmetric rolling and recovery/recrystallization. Generally, with an applied strain along [110] or [111], the C interstitial in ferrite is greatly stabilized, and can lead to an increase in the C concentration within the ferrite grains by up to two orders of magnitude, as mentioned in a report by Nematollahi et al. [33], which could likely provide the chemical potential for the rapid and local high-density precipitation. 

**Figure 11:**
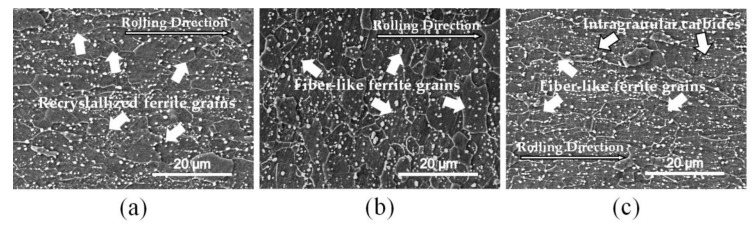
Scanning electron microscopy (SEM) morphology analysis of samples predeformed with a total reduction of 50% and reheated at 750 °C for 20 s in transverse section with (**a**) AR = 1.0, (**b**) AR = 1.2 and (**c**) AR = 1.5.

In Figure 11, the dispersed carbides in the transverse section can be detected in all the samples processed with different AR. Compared with the SEM images shown in Figure 9a,d, it can be inferred that the AR of asymmetric rolling and its total reduction may have an effect on the precipitation behavior of carbides during reversed transformation, although the thickness of samples was different and affected the heating rate and holding temperature to some extent. In addition, most of the ferrite grains were almost recrystallized in Figure 11a, while fiber-like deformed ferrite gains were still distinguished in Figure 11b,c. In addition, the volume fraction of carbide in the symmetrically prerolled samples was measured about 4.0%, which appeared to be less than that in Figure 11b,c, 4.7% and 6.8%, respectively. Therefore, the recrystallization of ferrite was inhibited by precipitation of carbide before the reversed transformation.

**Figure 12:**
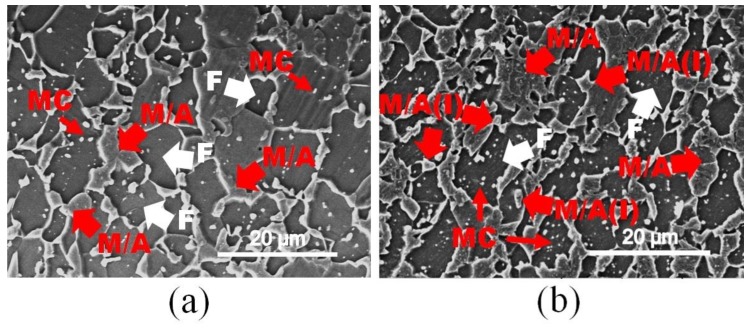
Scanning electron microscopy (SEM) morphology analysis of samples predeformed with a total reduction of 50% and reheated at 750 °C for 30 s in transverse section with (**a**) AR = 1.0 and (**b**) AR = 1.5.

As the holding time extended to 30 s at 750 °C, both reversed austenite transformation occurred in asymmetric prerolled samples with a reduction of 50%, as shown in Figure 12a,b. The volume of martensite transformed from austenite (M/A) increased to 51.2% with the increase of the AR, and the carbides were highly consumed at this moment, and all the remaining metal carbides (MCs) were located inside the ferrite grains. According to the M/A distributional morphology, the austenite grains preferred to nucleate at ferrite grain boundaries, as indicated with red arrows in Figure 12a. However, intragranular martensite transformed from austenite (M/A(I)) was also found in Figure 12b, which means that increasing the AR is beneficial to improve the nucleation rate during the reversed transformation.

**Figure 13:**
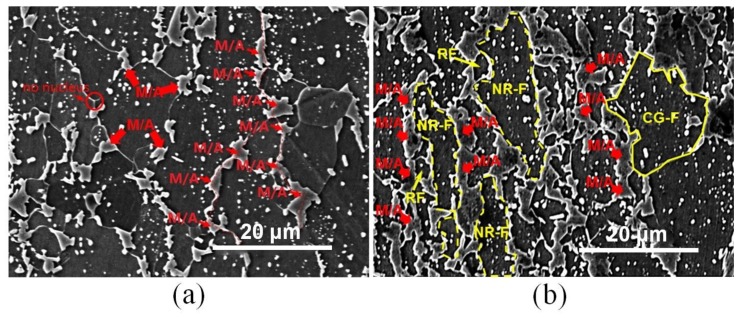
Scanning electron microscopy (SEM) morphology analysis of samples predeformed with a total reduction of 30% and reheated at 850 °C for 20 s in a longitudinal section with (**a**) AR = 1.0 and (**b**) AR = 1.2.

Additionally, both the 30% prerolled samples with AR = 1.0 and 1.2 were chosen to be reheated in a salt bath at a temperature of 850 °C, and the results are shown in Figure 13a,b, respectively. For the symmetrically prerolled sample, most of the RF grains had already grown to larger-size grains about 12 μm, while in the asymmetric prerolled sample, numerous non-recrystallized ferrite (NR-F) could be observed, and only a few RF grains and coarse grained ferrite (CG-F) were generated in the sample. Meanwhile, the volume of M/A in Figure 13a 15.5% was much less than that 43.6% in Figure 13b, which implies that the dynamics of reversed transformation in the asymmetrically prerolled samples was much faster. Moreover, the austenite grains preferentially nucleated on the ferrite grain boundaries according to the locations of the M/A, especially the triple junctions, which are typically present in Figure 13a. However, many M/A(I) were found in Figure 13b, indicating that a higher nucleation rate of reversed transformation could be achieved by asymmetric prerolling. 

## 4. Conclusions

In the current research, asymmetrically cryogenic rolling was used to prepare different initial microstructures in 22MnB5 steel. Ferrite recrystallization occurred prior to nucleation of austenite grains in the symmetrically prerolled samples, whereas the order was reversed in the asymmetrically prerolled samples. This means that ferrite recrystallization could be remarkably inhibited by asymmetric rolling at the LNT, while further precipitating of nanosized intragranular carbides consumed that strain-induced intrinsic energy during reheating and holding at 700–750 °C. Additionally, the nucleation of austenite inside the grains could be promoted by asymmetric prerolling and the dynamics of reversed transformation was also accelerated as the prestrain energy was greatly preserved and had a marked effect on such a process. Certainly, the reheating process should also be properly selected with a higher heating rate. Thus, it is important to fully understand the relationship among the predeformation, the reheating process and the commonly reversed microstructures, which can be helpful in the development of a new low-cost hot stamping process.

## Figures and Tables

**Table 1:** Chemical composition of 22MnB5 (wt %).

C	Si	Mn	P	S	Cr	Ti	B	Al	Fe
0.22	0.28	1.25	0.01	0.005	0.23	0.02	0.003	0.03	Bal.
